# A Facile Route to Large‐Area 2D Pt

**DOI:** 10.1002/advs.202517427

**Published:** 2025-11-28

**Authors:** Minsik Kong, Zhen Zhang, Weiyin Chen, Ethan Yupeng Zheng, Aubrey Penn, Ju Li

**Affiliations:** ^1^ Department of Nuclear Science and Engineering Massachusetts Institute of Technology Cambridge MA 02139 USA; ^2^ Department of Materials Science and Engineering Massachusetts Institute of Technology Cambridge MA 02139 USA; ^3^ MIT.Nano, Massachusetts Institute of Technology Cambridge MA 02139 USA

**Keywords:** 2D, adhesion layer, hydrogen‐evolution reaction, platinum, printing, sputtering

## Abstract

Platinum (Pt) is a popular hydrogen‐evolution reaction (HER) catalyst, yet its high‐cost limits industrial deployment. This is addressed by incorporating an oxygen‐deficient, gallium (Ga)‐rich gallium oxide (GaO_x_) adhesion layer that reverses the dewetting thermodynamics, yielding continuous 2D Pt at sub‐nanometer thickness by simple direct current (DC) sputtering. Alloy anchoring and vacancy chemisorption produce mechanically robust, transparent, conductive films with high thermal stability. During HER, 2D Pt/GaO_x_ reduces, forming a Ga‐Pt that further smoothens. The 1 nm film matches bulk Pt electrocatalytic activity while sustaining 1A cm^−2^ for 100 h without decay. Revealing the wetting mechanism including the effect of adhesion layer, and the depositing metals, the strategy generalizes to other noble metals with adhesion layers, offering a scalable route to ultrathin catalytic and electronic platforms.

## Graphical abstract

This work demonstrates a simple route to form continuous 2D platinum (Pt) films only up to 0.3 nm thick using a gallium oxide (GaO_x_) adhesion layer. The oxygen‐deficient GaO_x_ reverses wetting thermodynamics, enabling scalable sputtering of transparent, conductive, and robust 2D Pt. During hydrogen evolution, the films alloy with gallium (Ga), matching bulk Pt activity while sustaining long‐term stability.

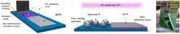

## 1 Introduction

Clean‐energy applications rely heavily on the development of robust catalysts.^[^ ^1^, ^2^ ^]^ Among these, platinum (Pt)‐based materials remain one of the most active and stable choices, for reactions like the hydrogen evolution reaction (HER).^[^ ^3^ ^]^ However, the high cost of bulk Pt necessitates research into alternative catalysts^[^ ^4^ ^]^ or strategies to utilize this precious metal more effectively.^[^ ^5^ ^]^ Key challenges include enhancing the Pt atom efficiency,^[^ ^6^ ^]^ improving mechanical durability against attacks like potential cycling or gas bubbling,^[^ ^7^, ^8^ ^]^ minimizing Pt loading^[^ ^9^ ^]^ without sacrificing performance, and developing scalable synthesis methods.^[^ ^10^ ^]^

Previous research has explored various nanostructured Pt or alloy catalysts for HER under different electrocatalytic conditions.^[^ ^11^, ^12^ ^]^ Well‐defined nanostructures like nanoparticles on carbon supports^[^ ^13^ ^]^ or ultrathin Pt decorated particles^[^ ^5^ ^]^ have shown excellent activity. However, integrating large‐area ultrathin metal layers onto diverse substrates with conventional techniques such as pulsed laser deposition (PLD), atomic layer deposition (ALD), or molecular beam epitaxy (MBE) demands highly specialized equipment and carefully controlled growth kinetics to fabricate nanostructures, yet, even with such precise processing, the solid‐state dewetting^[^ ^14^, ^15^ ^]^ issue persists unless an encapsulation layer is introduced. On common substrates like oxides or carbons, sputtered Pt adopts a Volmer‐Weber growth mode forming islands, raising resistance (electrocatalyst requires electronic percolation) and accelerating mechanical loss.

We address dewetting at its thermodynamic root by sputtering Pt onto an oxygen (O)‐deficient, metallic gallium (Ga) included gallium‐oxide (GaO_x_) adhesion layer obtained using liquid metal dewetting‐induced oxide printing method.^[^ ^16^ ^]^ The printed GaO_x_ was confirmed as non‐stoichiometric, and electrically conductive. The printed GaO_x_ was selected after considering both thermodynamic and kinetic factors governing Pt wetting. Ga provides sufficiently fast interfacial diffusion kinetics at the deposition temperature due to its near‐liquid nature, allowing rapid Pt‐Ga interaction during sputtering. This enabled robust adhesion onto various underlying substrates without extensive surface treatment.

**Figure 1:**
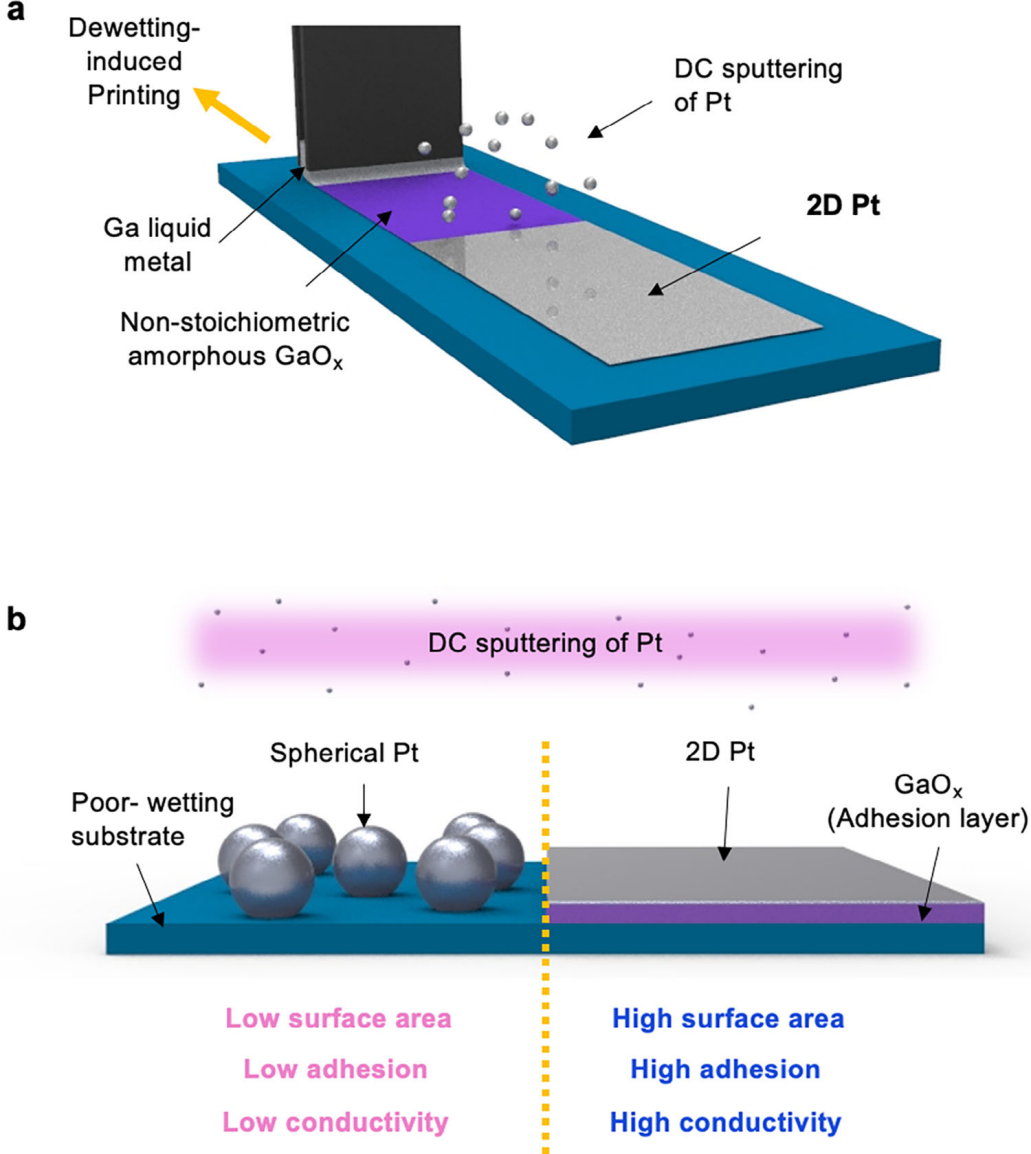
Schematics of 2D Pt fabrication and morphology with and without GaO_x_. a) Schematic of the 2D Pt fabrication process. Dewetting‐induced printing method was used to form a GaO_x_ adhesion layer on the desired substrate, followed by Pt deposition via DC magnetron sputtering. b) Schematic comparing the surface morphology of sputtered Pt with and without the GaO_x_ layer on a poor‐wetting substrate.

Here we aim to create ultrathin Pt films deposited directly onto large‐area printed GaO_x_ layers using simple direct current (DC) magnetron sputtering (**Figure** **1**a). With the GaO_x_ adhesion layer, even trace amount of Pt forms percolating film, rather than isolated islands or particles, having high surface area, strong adhesion and high electrical conductivity (σ ≈ 2.9 × 10^6^ S m^−1^ for 1 nm thick) (Figure 1b).

Transmission electron microscopy (TEM) combined with elemental mapping is performed to confirm in‐plane percolation compared to Pt deposited onto typical substrates like silicon with silicon oxide (Si/SiO_2_) wafers. Moreover, these thin Pt layers were stable under tape‐peel and cotton swab wipe tests, showing mechanical robustness and good adhesion to the substrates.

The wetting thermodynamics is estimated by the spreading coefficient *S*,^[^ ^17^, ^18^ ^]^ including three effects^[^ ^19^ ^]^ (i) a dispersion contribution (*S* ^d^), (ii) a chemisorption (Δ*W*_vac_) contribution from Pt‐O‐Ga bonding at O vacancies, and (iii) an alloy‐anchoring term (Δγ_alloy_) generated when residual metallic Ga in the as‐printed GaO_x_ reacts with arriving metal. The sign of *S* is the thermodynamic indicator where, *S* < 0 favors island (Volmer–Weber) growth, whereas *S* > 0 yields a continuous precursor film that can be sub‐nanometer in thickness and spreading like oil on water.^[^ ^20^ ^]^ The calculations reveal that *S* becomes positive (*S* > 0) only for the as‐printed GaO_x_ when the alloy‐anchoring term (iii) is included, explaining the formation of continuous Pt films. Extending the same analysis to other noble metals shows that only Pt and palladium (Pd) achieve *S* > 0, whereas gold (Au) and silver (Ag) remain *S* < 0, matching with the experiment.

Finally, this thin Pt layer was electrochemically tested as the catalyst for HER. Applying high negative potential (up to ‐1 V vs reversible hydrogen electrode (RHE)) in sulfuric acid condition (0.5 m H_2_SO_4_) etches and reduces the GaO_x_ and induces the formation of a gallium‐platinum (Ga‐Pt) alloy film that maintains ultra thinness. During the reaction, the Pt film got smoother without coarsening and exhibited excellent HER activity, with a Tafel slope of 54.4 (initial) and 44.0 mV dec^−1^ (after 100 h), and overpotentials of 56.5 mV at 10 mA cm^−2^ and 139.3 mV at 100 mA cm^−2^, comparable or even superior to bulk Pt (45.0 mV dec^−1^, 57.3, and 142.6 mV, respectively). Continuous HER operation over 100 h confirmed that the 2D Ga‐Pt is highly efficient and robust electrocatalytically.

## 2 Structural and Chemical Characterization of 2D Pt

**Figure 2:**
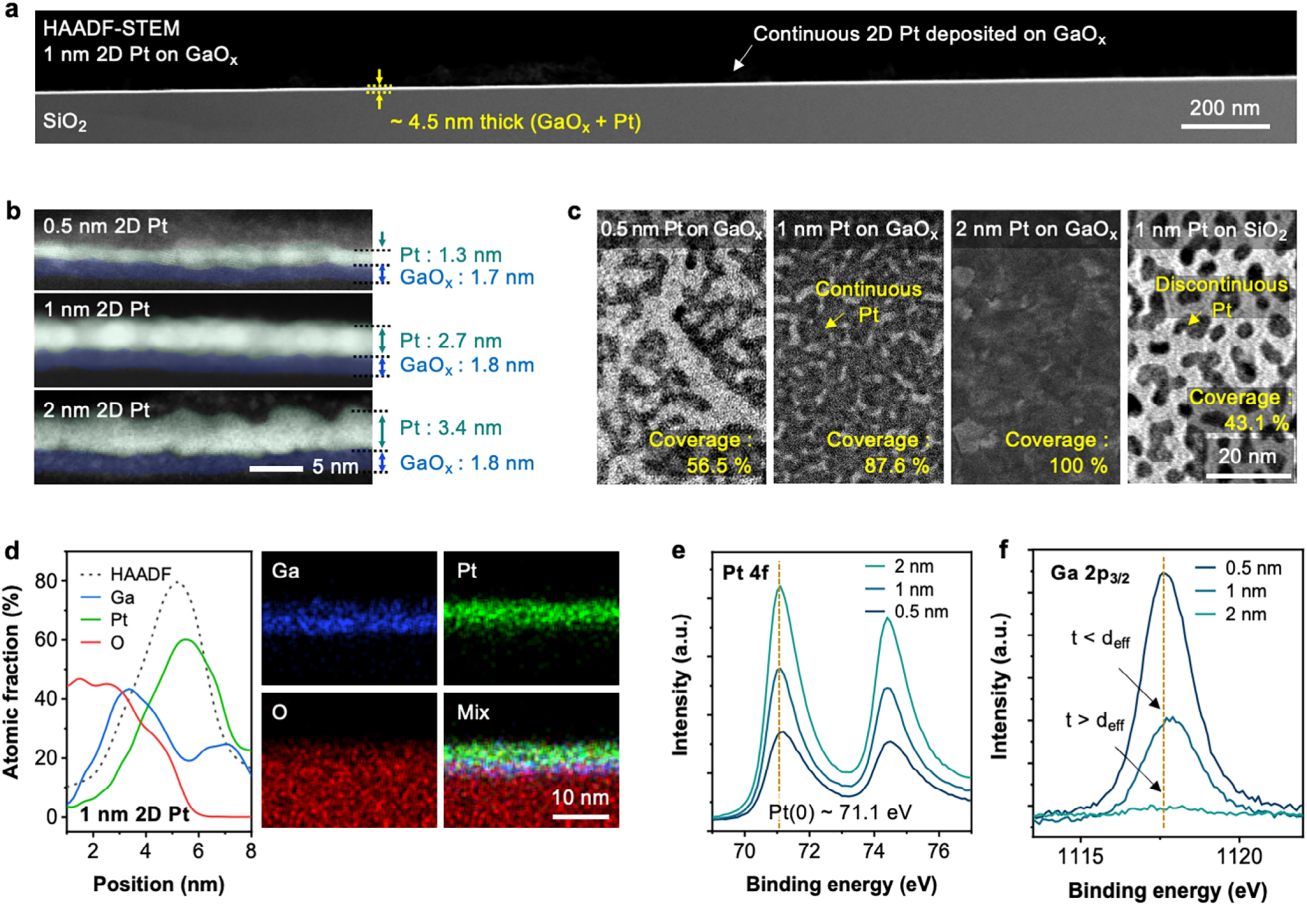
Morphological and chemical characterization of 2D Pt. a) Cross‐sectional STEM image of 1 nm 2D Pt on SiO_2_, demonstrating large‐area uniform coverage and a smooth, continuous film. b) Cross‐sectional STEM images of 2D Pt with varying Pt thicknesses. Note that thickness values are based on QCM measurements, which may differ slightly from actual thicknesses measured in the microscope. For clarity, Pt is colored green and GaO_x_ blue. c) Top‐view TEM images of 2D Pt at different Pt thicknesses, with 1 nm Pt on SiO_2_ shown as a reference. Pt coverage was quantified visually using image analysis software. d) EDS line scan and elemental mapping of 1 nm 2D Pt. e) Pt 4f XPS spectra of 2D Pt at different thicknesses. f) Ga 2p_3/2_ XPS spectra of 2D Pt at different thicknesses.

Scanning transmission electron microscopy (STEM) and transmission electron microscopy (TEM) images (**Figure** **2**a–c) illustrate the formation of continuous, high‐coverage 1‐nm thick 2D Pt networks on large‐area printed GaO_x_. Coverage up to inch‐wafer scale is further supported by electrical conductivity measurements, which will be discussed in detail later. Cross‐sectional STEM images in Figure 2b confirm well‐connected Pt films even at low nominal thicknesses ranging from 0.5 to 2 nm. To better visualize the atomic structure, a high‐resolution cross‐sectional high‐angle annular dark‐field (HAADF)‐STEM image with optimized contrast was obtained for the 1 nm 2D Pt film (Figure S1, Supporting Information). The atomic lattice fringes of Pt are clearly visible, confirming its crystalline nature and continuous morphology. The “thickness” here, corresponds to the value measured by a quartz crystal microbalance (QCM) during deposition, distinct from direct physical measurements through the microscope images. The discrepancy between these arises due to the contribution of in‐plane Pt coverage, confirmed by top view microscope images (Figure 2c). We validated the reliability of the QCM by depositing a thicker layer of Pt (20 nm), which closely matched the physical measurement (Figure S2, Supporting Information). Notably, the thickness of the printed GaO_x_ layer remains largely unchanged at ≈1.8 nm regardless of the deposited Pt amount, although its initial thickness was previously measured as 3.5 nm.^[^ ^16^ ^]^ This suggests that Pt slightly diffuses into the GaO_x_, forming a mixed interface (Figure 2c). As shown in Figure 2c, 0.5 nm 2D Pt shows roughly 2 nm‐width networks of Pt with 56.5% areal coverage. Increasing the thickness to 1 and 2 nm 2D Pt resulted in more extensive and uniform 2D Pt network, reaching 87.6% and 100% areal coverage, respectively. Notably, depositing 1 nm of Pt onto 100 nm SiO_2_ coated Si wafer yielded discontinuous Pt islands with 43.1% areal coverage, indicating poorer wetting property (Figure S3, Supporting Information).

Elemental analysis performed using energy‐dispersive X‐ray spectroscopy (EDS) and X‐ray photoelectron spectroscopy (XPS) (Figure 2d–f) provides additional insights into the surface composition and chemical state of Pt. The linear elemental profiles, together with EDS images, indicate a slight intermixing between Ga and Pt at the interface (Figure 2d). The XPS data for Pt 4f show a strong peak at 71.1 eV, characteristic of metallic Pt^0^, indicating that Pt remains metallic irrespective of the deposited thickness. The Ga 2p_3/2_ spectrum exhibits a broad peak centered at 1117.6 eV, suggesting the coexistence of Ga^0^ and Ga suboxides. The reduction of Ga 2p_3/2_ intensity with increasing Pt thickness (*t*) can be explained by electron scattering described by the inelastic mean free path (IMFP < 2 nm for aluminum K‐alpha (Al‐Kα) with Pt metal).^[^ ^21^ ^]^ When the Pt thickness is less than the effective probing depth (*d*_eff_ ≈ 3 × IMFP), Ga‐originating electrons maintain sufficient intensity. Conversely, for thicknesses exceeding this depth (*t* > *d*_eff_), electron scattering within the Pt film significantly reduces the Ga signal detected by XPS. This also confirms sufficient in‐plane Pt coverage of the underlying GaO_x_.

## 3 Electrical, Optical, and Mechanical Characterization of 2D Pt

**Figure 3:**
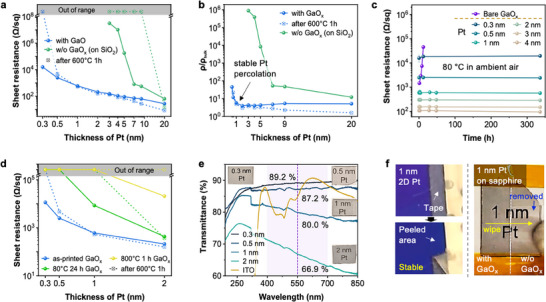
Electrical, optical and mechanical properties of 2D Pt. a) Sheet resistance of Pt films as a function of thickness of Pt on GaO_x_ and SiO_2_. Dashed lines represent values after annealing at 600 °C for 1 h. b) Normalized resistivity (ρ/ρ_0_, relative to bulk Pt) as a function of Pt thickness, highlighting stable Pt percolation starting around 1 nm on GaO_x_. c) Thermal stability of 2D Pt films deposited on GaO_x_ during annealing at 80 °C in ambient air, shown by changes in sheet resistance over time. d) Comparison of sheet resistance for Pt films deposited on GaO_x_ substrates prepared under various conditions (as‐printed and annealed at different temperatures). e) Optical transmittance spectra of 2D Pt films with different thicknesses. Commercial ITO film is included as a reference. The purple shaded band indicates the visible range (≈ 400–700 nm), while the dashed purple line marks the representative wavelength of 550 nm. f) Mechanical stability tests (tape‐peel and wipe tests) of 1 nm Pt film deposited on GaO_x_/sapphire substrate, demonstrating significantly improved adhesion compared to films without GaO_x_.

**Figure** **3**a compares the sheet resistance of Pt films deposited with and without GaO_x_ on Si/SiO_2_ wafer substrates as a function of Pt thickness. Pt films as deposited with GaO_x_ exhibited measurable sheet resistance even at thicknesses as low as 0.3 nm, whereas films on SiO_2_ (surface of Si/SiO_2_, 100 nm‐thick) substrates showed “out of range” high sheet resistance, until reaching a thickness of 3 nm with still very high sheet resistance. Even beyond 3 nm, Pt films with GaO_x_ consistently demonstrated lower sheet resistance compared to films on SiO_2_, indicating enhanced electrical continuity. After annealing at 600 °C for 1 h, the samples with Pt thicknesses higher than 1 nm further improved, likely due to the relaxation of internal stress and grain growth. The sheet resistance of 0.3 nm 2D Pt layer became insulating, and the 0.5 nm thick layer showed a slight increase in resistance. After annealing, Pt deposited on SiO_2_ lost electrical conductivity even at a thickness of 9 nm, which is expected to be described as solid‐state‐dewetting.^[^ ^15^ ^]^ In addition, a comparison with the conventional titanium (Ti) adhesion layer further confirmed the superior electrical continuity, lower percolation threshold, and enhanced thermal stability of the GaO_x_ adhesion layer (Figure S4, Supporting Information). This demonstrates that GaO_x_ not only promotes early percolation of ultrathin Pt films but also suppresses interfacial degradation during thermal processing, outperforming the commonly used Ti adhesion layer.

Further looking into the resistivity of the 2D Pt, Figure 3b plots the resistivity ratio (ρ/ρ_bulk_) as a function of Pt thickness, highlighting stable metallic percolation at ≈1 nm with the GaO_x_ adhesion layer. Beyond the thickness 1 nm, resistivity values stabilize and approach near‐bulk values (ρ/ρ_bulk_ = 1) after annealing, demonstrating excellent electrical performance.

The small residual gap between the measured and theoretical bulk resistivity even for large thicknesses is typical for sputtered Pt which depends largely on the deposition conditions and pre‐ or post‐treatments.^[^ ^22^ ^]^ It is noteworthy that the resistivity ratio remains remarkable at thicknesses below 10 nm, despite the anticipated significant increase in electron‐surface scattering as the film thickness approaches or becomes much smaller than the bulk electron mean free path (λ_e_≈10 nm for bulk Pt).^[^ ^23^ ^]^ The thickness‐dependent resistivity was compared with the Fuchs–Sondheimer (FS) model accounting for electron surface and interface scattering (Figure S5, Supporting Information) to examine the influence of scattering more quantitatively. The inclusion of these scattering effects reproduces the deviation from the bulk value well, indicating that the observed behavior is primarily governed by conventional size‐effect scattering rather than by any anomalous transport mechanism.

The long‐term thermal stability of 2D Pt films under mild 80 °C annealing in ambient air is presented in Figure 3c. All samples maintain stable electrical performance even after 300 h, with negligible variation in the sheet resistance. In contrast, bare GaO_x_ without Pt coating shows higher and unstable sheet resistance which eventually turns insulating, confirming that metallic Pt is the dominant reason for the high, stable electrical conductivity. An aspect ratio approaching 10^8^ is the reason we name it 2D Pt.

In Figure 3d, the impact of the initial chemical composition of underlying GaO_x_ adhesion layer, according to thermal annealing is investigated. The initial state of GaO_x_ was controlled by the post‐annealing condition of printed GaO_x_ following the previous reported paper (non‐stoichiometric amorphous GaO_x_ with metallic Ga inclusion for as‐printed sample, non‐stoichiometric amorphous without Ga inclusion after 80 °C for 24 h, and stoichiometric polycrystalline after 800 °C for 1 h).^[^ ^16^ ^]^ Percolation threshold increased for 80 °C, 24 h GaO_x_ (1 nm), and even more for 800 °C, 1 h GaO_x_ (2 nm). Thermal annealing under 600 °C, 1 h made both Pt film deposited on pre‐annealed GaO_x_ lose its conductivity, showing lower thermal stability compared to the one deposited on as‐printed GaO_x_.

The optical properties of the samples were characterized by ultraviolet–visible (UV‐vis) spectroscopy, as shown in Figure 3e. Samples were prepared on *c*‐plane sapphire substrates, with a bare sapphire wafer as a baseline correction. Owing to their ultrathin nature relative to the optical skin depth of Pt (≈20 nm),^[^ ^24^ ^]^ the 2D Pt layers remain highly transparent, exhibiting transmittances of 89.2%, 87.2%, 80.0%, and 66.9% at 550 nm for nominal Pt thicknesses of 0.3 nm, 0.5, 1, and 2 nm, respectively (indicated by the purple dotted line), comparable to the indium–tin–oxide (ITO) reference. Inset digital images of the actual samples placed over printed text visually confirm the high transparency across all thicknesses. Unlike ITO, which displays pronounced peaks and dips due to interband transitions and epsilon‐near‐zero feature,^[^ ^25^ ^]^ the ultrathin Pt films exhibit a nearly linear, color‐neutral transmittance across the visible spectrum (indicated by the purple band), consistent with a Drude‐type free‐electron behavior.^[^ ^26^ ^]^

Figure 3f illustrates the mechanical robustness and strong adhesion of ultrathin 1 nm 2D Pt films deposited onto Si/SiO_2_ wafer, and sapphire substrate. A tape‐peel test (left panel) confirms excellent adhesion, with no observable delamination of the Pt film, indicating strong bonding at both GaO_x_‐Pt, and GaO_x_‐SiO_2_ interface. Additionally, a cotton‐swab wiping test (right panel) clearly demonstrates the mechanical stability of the 2D Pt film, as the film remains intact, whereas the portion without GaO_x_ is completely removed (Movie S1, Supporting Information). The robustness of the 2D Pt could be also observed while transferring the film on TEM grid as in Movie S2 (Supporting Information). As in the movie, the free‐standing 1 nm 2D Pt/ GaO_x_ film can be bent and withstand moderate strain applied with a tweezer while immersed in deionized water. These observations confirm that the printed GaO_x_ layer significantly enhances the mechanical durability of ultrathin 2D Pt films and the adhesion to the underlying substrates.

## 4 Wetting Mechanism of Pt on Printed GaO_x_

The wetting behavior of Pt highly relies on the chemistry of the underlying GaO_x_. As‐printed GaO_x_ has metallic Ga inclusion with high density of oxygen‐vacancies. Mild annealing at 80 °C for 24 h removes most metal Ga but preserves a high density of oxygen‐vacancies. After 800 °C crystallization step the surface becomes more stoichiometric beta‐gallium oxide (β‐Ga_2_O_3_) with few vacancies. The spreading coefficients *S* for different initial state of GaO_x_ with the SiO_2_ as a reference are calculated sequentially adding three contributions: (i) surface and interfacial dispersion, (ii) oxygen‐vacancy chemisorption of Pt on Ga^δ+^ sites (Pt‐O‐Ga bonds) and (iii) alloy anchoring (Pt‐Ga interlayer, reactive wetting) (Note S1, Supporting Information). Calculation shows that with the only contribution (i) or even adding (ii), *S* remained negative (*S* < 0), indicating that the Pt metals prefer isolated particle formation rather than a continuous film. Adding contribution (iii) ultimately switched S to positive for as‐printed GaO_x_, demonstrating that formation of an interfacial metal‐alloy anchor markedly improves wetting and enables thin‐film growth sufficiently for a continuous film at only 0.3 nm which well matches with the experimental data discussed in Figures 2 and 3.

To further investigate the wetting mechanism, S for three other noble metals (Pd, gold (Au), and silver (Ag)) are also investigated along with the Pt, all fabricated on as‐printed GaO_x_ with the same deposition conditions (Note S2, Supporting Information). Similarly, adding contribution (iii) switched *S* to positive for Pt and Pd, matching with the experimental results (Figure S6, Supporting Information).

## 5 Hydrogen Evolution Reaction (HER) Using 2D Pt

**Figure 4:**
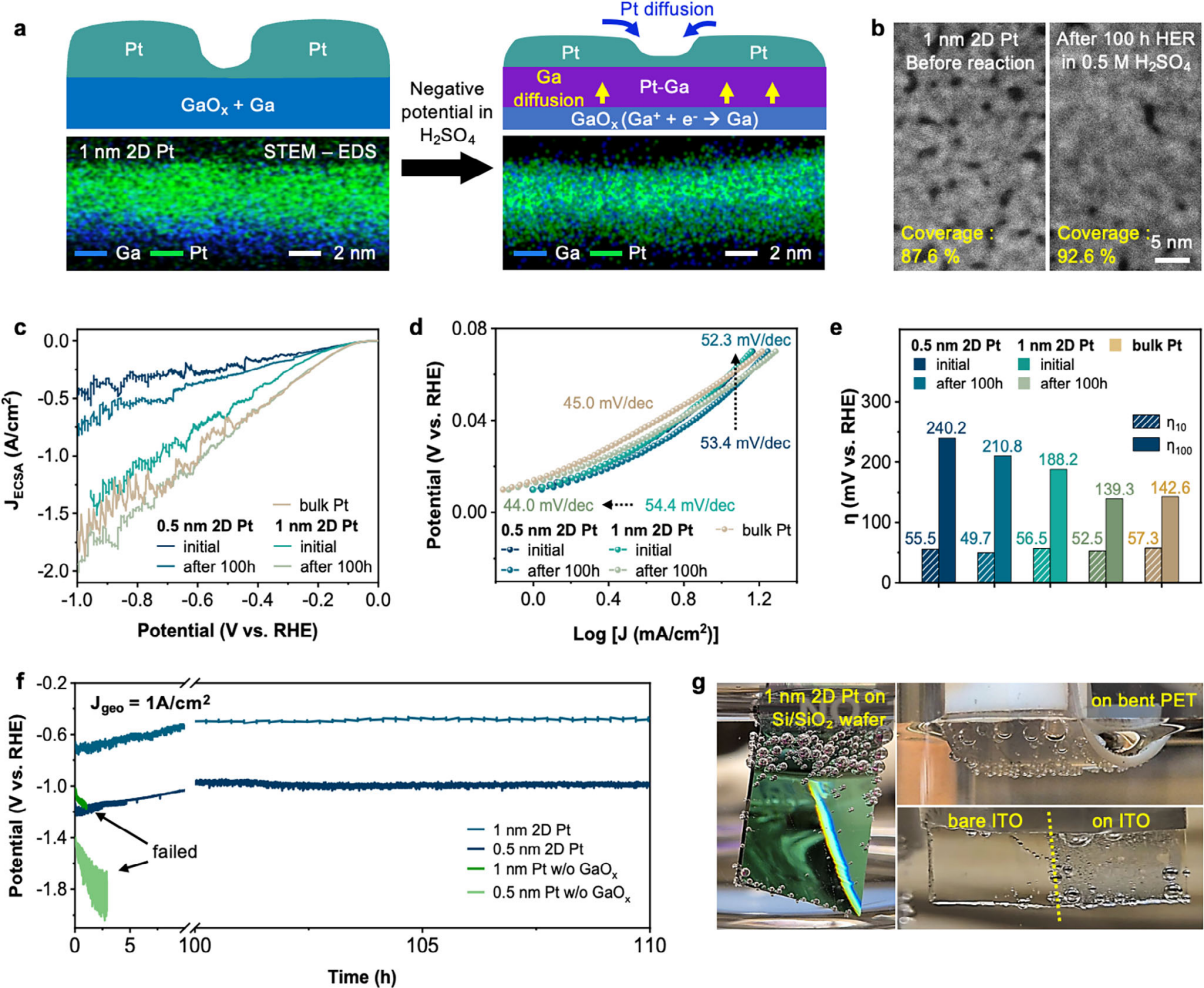
Hydrogen evolution reaction (HER) using 2D Pt. a) Schematic images of cross‐sections (top) and corresponding cross‐sectional STEM–EDS maps (bottom) of 1 nm 2D Pt film before and after 100 h HER in 0.5 M H_2_SO_4_. b) Top‐view STEM images of the same 1 nm 2D Pt film before and after the HER. c) ECSA‐normalized current density versus potential plotted LSV curve for 0.5 nm and 1 nm 2D Pt before and after 100 h, compared with a bulk Pt as a reference. d) Tafel plots for each sample. e) Overpotential comparison of each sample at 10 mA cm^−2^ (η_10_), and 100 mA cm^−2^ (η_100_). f) Long‐term chronopotentiometry at a geometric current density of 1 A cm^−2^ in 0.5 m H_2_SO_4_. g) HER of 1 nm 2D Pt on various substrates (on Si/SiO_2_ wafer, Polyethylene terephthalate (PET), and indium‐tin‐oxide (ITO)).

**Figure** **4**a illustrates the elemental reconstruction of 1 nm 2D Pt electrode after long‐term 100 h HER reaction, with cross‐sectional STEM‐EDS map. Strong negative potential applied to the working electrode in 0.5 m H_2_SO_4_ induces etching of exposed GaO_x_, and unexposed GaO_x_ reduced back to Ga metal (Ga^3+^ + 3e^−^ ⇌ Ga(s) at −0.56 V).^[^ ^27^ ^]^ Ga atoms diffuse upward and intermix with the 2D Pt, forming an ultrathin interfacial Pt‐Ga alloy layer^[^ ^28^ ^]^ as confirmed by the elemental analysis using XPS (Figure S7, Supporting Information). This interfacial alloying slightly perturbs the Pt 5d electronic structure, enhancing interfacial adhesion and stability without inducing dewetting or loss of electrocatalytically active Pt sites. High‐resolution HAADF‐STEM images (Figure 4b) of the 1 nm 2D Pt sample after 100 h HER show a more percolated network which initially showed 87.6 % coverage (left), to 92.6 % without any dewetting–induced coarsening (right) (Figure S8, Supporting Information). Immersing the 2D Pt electrode in 0.5 m H_2_SO_4_ for 100 h under open‐circuit conditions produced only a slight increase in sheet resistance, demonstrating its intrinsic chemical stability (Figure S9, Supporting Information).

Linear sweep voltage (LSV) curve for HER with current densities normalized with the electrochemical surface area (*J*_ECSA_) are plotted for 0.5 and 1 nm 2D Pt films, both initially and after 100 h of HER operation, together with a bulk Pt as a reference (LSV with geometric area, mass activity and Nyquist plot in Figures S10 and S11, Supporting Information). The 2D Pt samples and the bulk Pt reference depositing 20 nm with the same sputtering condition were prepared on glassy carbon as a back‐substrate to reduce the lateral resistance. The 2D Pt exhibit comparable currents with the bulk Pt even slightly exceeding with 1 nm 2D Pt after 100 h. In addition to the general activation process of the Pt surface,^[^ ^29^ ^]^ the reconstruction of Pt atoms,^[^ ^30^, ^31^, ^32^ ^]^ alloying with Ga,^[^ ^28^ ^]^ etching of insulating GaO_x_,^[^ ^33^ ^]^ and reduction of GaO_x_ back to metallic Ga are all likely the causes of this.

Tafel plots (Figure 4d) and overpotential value (Figure 4e) clearly indicate the change for 0.5 and 1 nm 2D Pt before and after the 100 h durability test. The initial Tafel slope for 0. and 1 nm 2D Pt showed 53.4 and 54.4 mV dec^−1^, respectively, slightly lower than bulk Pt reference (45.0 mV dec^−1^). After 100 h reaction, the slope changed to 52.3 and 44.0 mV dec^−1^, which 1 nm 2D Pt slightly exceeding the bulk Pt reference. The overpotential at current density 10 mA (η_10_) drops from 55.5 to 49.7 mV for the 0.5 nm Pt film and from 56.5 to 52.5 mV for the 1 nm Pt film after 100 h HER. Both values surpass the 57.3 mV bulk Pt reference, emphasizing that atomic layer thin catalysts can meet or exceed the intrinsic catalytic kinetics of their thick‐film counterparts. The overpotential at 100 mA (η_100_), which is often regarded as an industrial benchmark, shows 139.3 mV for 1 nm 2D Pt after 100 h, comparable with the bulk reference.

Chronopotentiometry recorded at the geometric current density (*J*_geo_) of 1 A cm^−2^ more than 100 h demonstrates outstanding operational robustness (Figure 4f). The 1 nm and 0.5 nm 2D Pt initially show an improvement of potential, matching with the previously discussed results. Whereas identically sputtered Pt layers directly on glassy carbon delaminate within the first few hours for all samples. The result highlights the crucial role of the GaO_x_ wetting/anchoring layer in preventing film dewetting and sustaining catalytic activity under vigorous gas evolution.

Figure 4g demonstrates the HER of 1 nm 2D Pt on various substrates, including on Si/SiO_2_ wafer (LSV in Figure S12, Supporting Information), polyethylene terephthalate (PET), and ITO. Note that the Si/SiO_2_ wafer and PET substrate are electrically insulating (350 nm‐thick SiO_2_), so electron transport for HER occurs exclusively through the ultrathin 2D Pt film. Consequently, HER activity is highest near the electrode contact region and gradually decreases with distance, due to potential drops along the high sheet resistance of the 2D Pt. This behavior, also seen in Figure 4g and Figure S9 (Supporting Information), highlights the importance of contact engineering when employing insulating supports. Due to its thin nature, the 2D Pt on PET could be deformed by heating above the glass‐transition temperature (≈100 °C) of the PET without losing electrical percolation with modest increase in sheet resistance (754.1 to 1648.5 Ω sq^−1^), while preserving HER activity. The representative transparent conductive oxide ITO could be tuned to show HER by coating the 2D Pt@GaO_x_ on the surface with minor transparency decrease, for viewing the H_2_ bubble dynamics (Movie S3, Supporting Information).

## 6 Conclusion

This study demonstrates that the non‐stoichiometric, metallic GaO_x_ adhesion layer enables the formation of continuous, ultrathin 2D Pt. Spontaneous Pt‐Ga alloy anchoring, combined with oxygen vacancy‐assisted chemisorption, yields electronically percolating, optically transparent, and mechanically robust 2D Pt, capable of withstanding bending, tape‐peel, ​swabbing, and even free‐standing in water. These properties are retained even after high‐temperature annealing and under harsh HER operations that typically degrade sputtered metals.

Under cathodic bias, GaO_x_ reduces and alloys, smoothing the film, enhancing utilization, and lowering both Tafel slopes and overpotential. A 1 nm film slightly outperforms bulk Pt while using a fraction of the precious metal, sustaining 1 A cm^−2^ for over 100 h without degradation.

As the wetting, and thermal stability mechanism is governed by a metal‐rich, vacancy‐rich template with the chemistry of metal, the strategy should extend to other scarce metals (iridium (Ir), ruthenium (Ru)) and bimetallic catalysts, enabling ultrathin, flexible, transparent conductors that unite high conductivity with mechanical resilience. Thus, GaO_x_ functions here as a transient, self‐regulating interlayer that maximizes atom efficiency, enabling scalable path to cost‐effective, large‐area catalytic and optoelectronic platforms.

## 7 Experimental Section

### Materials

Gallium (Ga, 99.999%) was purchased from Indium Corp, platinum (Pt, 99.99%), and palladium (Pd, 99.99%) metal targets were from AJA International. Silicon (Si) wafers, and sapphire wafers are purchased from University Wafer. Potassium chloride (KCl, ≥ 99.0%) was from Sigma‐Aldrich. Polyacrylic acid (PAA, 450k), poly(methyl methacrylate) (PMMA, 350k) polymers which were used for the substrates were from Sigma‐Aldrich. Indium tin oxide (ITO, R< = 14 ohm sq^−1^, 115 nm) was from Welljoin. Polyethylene terephthalate (PET) was from CalPalmy. Molybdenum (Mo) slot transmission electron microscope (TEM) grid Ted Pella, Polishing supplies Allied. Isopropanol, and acetone (99.5%) solvents were from Avantor VWR. Sulfuric acid (H_2_SO_4_) 2.0 N in aqueous solution was from Avantor VWR and used after diluting. Electrochemical three electrode cell setup including the counter carbon rod electrode, reference silver/silver chloride (Ag/AgCl) electrodes are purchased from Stonylabs. The Sigradur G Glassy (Vitreous) Carbon Plate were from SPI supplies.

### Dewetting‐Induced Gallium Oxide (GaO_x_) Printing

The printing setup and conditions were established based on a previously reported paper.^[^ ^16^ ^]^ The printer head was fabricated by attaching two glass slides with a 1 mm gap. Ga metal sources were prepared by melting pellets at ≈60 °C and injecting it into the printer head. The printer head containing the liquid Ga was then brought close to the target substrate until the liquid Ga meniscus contacted the substrate. Printing was performed by translating either the printer head or the substrate at the desired speed.

All substrates, including silicon wafers, c‐plane sapphire, and various polymers, were solvent‐cleaned sequentially with acetone, isopropyl alcohol (IPA), and deionized (DI) water prior to use.

### Facile Method to Fabricate 2D Pt

GaO_x_ samples were printed on a target substrate. As‐printed GaO_x_ sample was stored inside an argon (Ar)‐filled glove box immediately after printing, to minimize the oxidation. 80 °C, 24 h annealed GaO_x_ were prepared in advance and simultaneously deposited the metal with the as‐printed GaO_x_. The metal deposition was carried out using direct current (DC) magnetron sputtering (AJA Model ATC Orion 5) at a rate of 0.5 Å s^−1^ and a working pressure of 3 × 10^−3^ Torr, with 12 sccm of Ar gas continuously purged into the chamber. Prior to deposition, the chamber was pumped down to a base pressure below 3 × 10^−5^ Torr. During deposition, the stage was rotated without applying additional heating. The thickness values reported in this work were estimated using a quartz crystal microbalance (QCM) inside the sputtering chamber and validated by cross‐sectional TEM measurements.

### GaO_x_ Transfer Method for TEM Characterization

The 2D Pt‐deposited oxide was transferred by introducing a polymer sacrificial layer.^[^ ^34^ ^]^ The GaO_x_ was printed onto a water‐soluble PAA film. The PAA substrate was prepared by spin‐coating a 10 wt.% PAA solution in DI water onto a glass slide at 3000 rpm, followed by annealing at 80 °C for 1 h. After Pt deposition, the entire sample was immersed in DI water to dissolve the PAA layer. Upon dissolution of the PAA, the free‐standing 2D Pt/GaO_x_ film floated to the surface of the water. The floating 2D Pt/GaO_x_ film was then gently scooped using a TEM grid (Movie S2, Supporting Information). The DI water was allowed to evaporate at room temperature for 24 h. To improve adhesion and remove surface contaminants, the transferred sample on the TEM grid was further annealed at 70 °C for 2 h in a vacuum oven right before TEM characterization.

### Three‐Electrode Cell Setup for Hydrogen Evolution Reaction (HER) Test

HER measurements were performed using a three‐electrode cell setup. The reference electrode was an Ag/AgCl electrode prepared with saturated KCl solution. A carbon rod served as the counter electrode to prevent Pt redeposition onto the working electrode.^[^ ^35^ ^]^ The working electrode, 2D Pt, was directly fabricated on a flat glassy carbon substrate. Electrochemical tests were carried out in 0.5 m H_2_SO_4_ electrolyte at room temperature (≈25 °C). Electrochemical surface area (ECSA) of the samples was estimated by measuring the cyclic voltammetry (CV) curves at various scan rates in a non‐Faradaic potential window.^[^ ^36^ ^]^ The double‐layer capacitance (C_dl_) was determined from the slope of the linear fit of the capacitive current versus scan rate plot, with a specific capacitance value (C_s_) of a smooth poly‐Pt surface (35 µF cm^−2^).^[^ ^37^ ^]^ The cell did not include any stirring or gas purging system to avoid disturbance of the ultrathin Pt films; however, electrochemical impedance spectroscopy (EIS) analysis (Figure S11, Supporting Information) confirmed negligible mass transport resistance under these conditions.

### Characterization

This work was carried out in part using MIT.nano facilities. Scanning transmission electron microscopy (STEM) and TEM imaging were performed using a Themis Z G3 Cs‐corrected S/TEM and a Talos F200i TEM at MIT.nano, MA, USA. Cross‐sectional TEM specimens were prepared by mechanical polishing (Multiprep, Allied) followed by ion milling (Fischione 1051 TEM mill)^[^ ^38^ ^]^ to avoid Ga‐beam damage commonly observed with focused ion beam (FIB) preparation.^[^ ^39^ ^]^ Sheet resistance was measured using a four‐point probe system (Jandel Model RM3‐AR). Optical transmittance of the samples was measured with a UV‐vis spectrophotometer (PerkinElmer Lambda 1050) after subtracting the transmittance of the quartz substrate. Chemical composition and electronic binding states were analyzed using X‐ray photoelectron spectroscopy (XPS, PHI VersaProbe II X‐ray Photoelectron Spectrometer). Electrochemical data were collected and analyzed with EC‐Lab software. Pt coverage measuring is done by ImageJ software.

## Conflict of Interest

The authors declare no conflict of interest.

## Supporting information



Supporting Information

Supplemental Video 1

Supplemental Video 2

Supplemental Video 3

## Data Availability

The data that support the findings of this study are available in the supplementary material of this article.
