# Supplementary material for: A Facile Route to Large‐Area 2D Pt
Source: Adv Sci (Weinh). 2025 Nov 28;13(8):e17427. doi: 10.1002/advs.202517427 (PMC12884711; doi:10.1002/advs.202517427)
Supplement: Supplementary file 1 — Supporting Information [file ADVS-13-e17427-s001.docx]

Supporting Information

A Facile Route to Large-Area 2D Pt

Minsik Kong, Zhen Zhang, Weiyin Chen, Ethan Yupeng Zheng, Aubrey Penn, Ju Li*

Minsik Kong, Weiyin Chen, Ju Li

Department of Nuclear Science and Engineering, Massachusetts Institute of Technology, Cambridge, MA 02139, USA

Department of Materials Science and Engineering, Massachusetts Institute of Technology, Cambridge, MA 02139, USA

Email: [liju@mit.edu](mailto:liju@mit.edu)

Zhen Zhang, Ethan Yupeng Zheng

Department of Materials Science and Engineering, Massachusetts Institute of Technology, Cambridge, MA 02139, USA

Aubrey Penn

MIT.Nano, Massachusetts Institute of Technology, Cambridge, MA 02139, USA

**Table of Contents**

1. **Notes ---------------------------------------------------------------------------------------------------** 3

- Note S1. Wetting dynamics of Pt on different initial chemical composition of GaO_x_
- Note S2. Wetting dynamics with different metals on as-printed GaO_x_

1. **Supporting Figures ----------------------------------------------------------------------------------** 7

- Figure S1. Cross-sectional HAADF-STEM image of the 1 nm 2D Pt film on GaO_x_.
- Figure S2. Cross-sectional STEM-HAADF and EDS images of the 20 nm-thick sputtered Pt on GaO_x_
- Figure S3. Pt surface coverage calculation, with and without GaO_x_ using software-processed mask image.
- Figure S4. Sheet resistance data for Pt film on GaO_x_, SiO_2_, and Ti adhesion layers.
- Figure S5. Comparison of thickness-dependent resistivity of Pt films with the Fuchs–Sondheimer (FS) model.
- Figure S6. Sheet resistance data for other noble metals (Pd, Au, Ag) deposited on GaO_x_.
- Figure S7. XPS spectra before and after HER for 100h.
- Figure S8. Pt surface coverage calculation, before and after 100h HER, using software-processed mask image.
- Figure S9. Sheet resistance of 2D Pt in 0.5 M H₂SO₄ for 100 h under open-circuit condition.
- Figure S10. LSV with current density by geometric area (J_geo_) and Pt mass loading (mass activity).
- Figure S11. Nyquist plot for tested samples.
- Figure S12. LSV of 1 nm 2D Pt on Si/SiO₂ substate.

1. **Movies ------------------------------------------------------------------------------------------------** 16

- Movie S1. Wipe test with 1 nm Pt on sapphire substrate with, and without GaO_x_ adhesion layer
- Movie S2. Free-standing 1 nm 2D Pt with GaO_x_ transferring to TEM grid.
- Movie S3. Transparent 2D Pt HER demonstration on ITO substrate

1. **References -------------------------------------------------------------------------------------------** 17

1. Notes

**Note S1. Wetting dynamics of Pt on different initial chemical composition of GaO_x_**

Wetting dynamics for sputtered Pt on different initial state of GaO_x_ is evaluated by calculating the spreading coefficient (S). The metal prefers to be wet and form a film when S > 0, and dewet, forming isolated particles when S < 0. The total spreading coefficient (S^total^) will be calculated adding the effects of i) surface & interfacial dispersion effect (S^d^), ii) oxygen-vacancy chemisorption effect (ΔW_vac_), and iii) alloy-anchoring effect (Δγ_alloy_). Although the total work of adhesion generally includes both dispersion and polar contributions, we focus on dispersion and chemisorption (oxygen-vacancy) terms here, since the sputtered GaOₓ is amorphous and highly defective, and noble metals like form only weak polar interactions with the oxide. The dominant wetting mechanisms are therefore governed by van der Waals forces, vacancy chemisorption, and alloy formation.

It should be noted that this model considers the dominant dispersion, vacancy chemisorption, and alloy-anchoring terms to provide a simplified yet physically meaningful estimation. Other possible contributions such as polar (acid–base) interactions, interfacial strain, substrate roughness, and deposition-related effects were not explicitly included, as the aim here is to capture the qualitative wetting trend and sign change of S, rather than to achieve exact numerical accuracy. Values used for the calculation were from previous reported papers. Symbols used for the calculation are listed as followed.

| **Symbol** | **Meaning** |
| --- | --- |
| γ_LV_ = γ_M_ | free-surface energy of the liquid–vapor interface, metal (Pt, Pd, Au, Ag, Ga) |
| γ_SV_ | free-surface energy of the substrate (GaO_x_) |
| γ_SL_ | liquid-substrate interfacial energy (Pt-GaO_x_) |
| W_ad_ | work of adhesion (Dupré) |
| S | spreading coefficient |
| t_c_ | calculated electrical percolation thickness |
| L, S, V | liquid (metal), solid (substrate), vapor (air) respectively. |

i) Spreading coefficient only for dispersion effect (S^d^) could be calculated using the equations,^[1]^


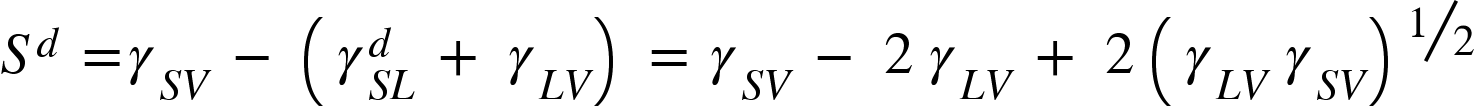


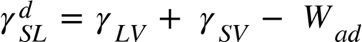


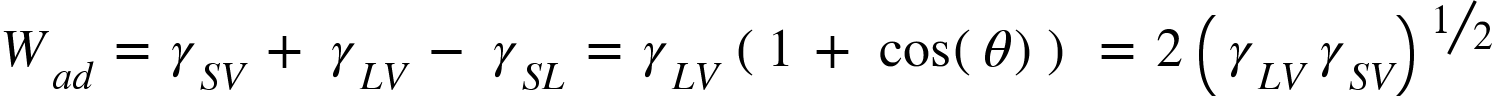


ii) Oxygen-vacancy chemisorption (ΔW_vac_) adhesion effect^[2]^ could be calculated using the equation,


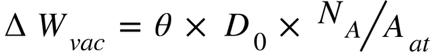


θ = fractional surface-site vacancy coverage

D_0_ = gas-phase M-O bond-dissociation energy (chemical adhesion)

N_A_ / A_at_ : converts per-atom energy to J m^-2^ (1 eV atom^-1^ = 1.068 J m^-2^)

A_at_ = 1.50 × 10^-19^ m² for close-packed Pt(111)

iii) Alloy-anchoring effect (Δγ_alloy_) could be calculated using following equation,


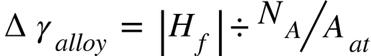


ΔH_f_ = Enthalpy of alloy formation. chemical potential of the alloy

Here are the values from references used for the calculation.

| **Metal** | **θ**^[3]^ | **D₀ (eV)^[^**^4]^ |
| --- | --- | --- |
| Pt | 0.42 | 4.01 |

| **Alloy** | **ΔH_f_ (kJ mol⁻¹ atoms)** | **Δγ_alloy_ (J m⁻²)** |
| --- | --- | --- |
| Pt-Ga | − 185 | + 2.05 |

|  | **O-vacancy fraction (θ)^[^**^3]^ | **Metal inclusions^[^**^3]^ | **γ_SV_ (J m⁻²)** |
| --- | --- | --- | --- |
| γ_Pt_ | - | - | 2.055^[5]^ |
| as-printed GaOₓ | 0.42 | Yes | 0.10^[6]^ |
| 80 °C / 24h GaOₓ | 0.33 | No | 0.22^*^ |
| 800 °C / 1h GaOₓ | 0.27 | No | 0.34^[7]^ |
| SiO_2_ reference | 0.00 | No | 0.30^[8]^ |

^*^Assumed to be average of as-printed and 800 °C annealed sample since amorphous materials varies a lot, which makes it hard to specify the value.

Mild annealing at 80 °C for 24h removes most of the metallic Ga but remains oxygen vacancies. Once the Ga interlayer is largely gone, Pt wets the surface mainly by chemisorption. After an 800 °C, 1h crystallization, the surface converts to stoichiometric β-Ga_2_O_3_ further decreasing the vacancies.

The calculated results are as followed.

| **Substrate** | **S^d^ (J m⁻²)** | **ΔW_vac_ (J m⁻²)** | **Δγ_alloy_ (J m⁻²)** | **S^total^ (J m⁻²)** |
| --- | --- | --- | --- | --- |
| as-printed GaOₓ | − 3.103 | + 1.80 | + 2.05 | + 0.665 |
| 80 °C / 24 h GaOₓ | − 2.545 | + 1.41 | 0 | – 1.14 |
| 800 °C / 1 h GaOₓ | − 2.098 | + 1.16 | 0 | – 0.94 |
| SiO_2_ reference | − 2.240 | 0 | 0 | – 2.24 |

Calculated S tells the main mechanism of 2D Pt formation is due to the metallic inclusion inside the GaO_x_ adhesion layer for as-printed GaO_x_.

**Note S2. Wetting dynamics with different metals on as-printed GaO_x_**

Wetting dynamics for various sputtered metals on as-printed GaO_x_ is calculated here. We sequentially added the three effects.

## i) Surface & interfacial dispersion effect (S^d^)

| **Quantity** | **Value (J m⁻²)^*^** |
| --- | --- |
| γ_Pt_ | 2.055^[5]^ |
| γ_Pd_ | 1.376^[5]^ |
| γ_Au_ | 1.345^[5]^ |
| γ_Ag_ | 1.046^[5]^ |
| γ_Ga_ | 0.718^[5]^ |
| γ_GaOx_ (as-printed) | 0.1^[6]^ |

**^*^**Note that the surface energy values used here corresponds to the liquid state of the metals, since near-atomically deposited metal is often treated as a pseudo-liquid.^[9,10]^

Calculated results are as followed.

| **Metal** | **W_ad_** | **γ^d^_SL_** | **S^d^** |
| --- | --- | --- | --- |
| Pt | 0.907 | 1.2488 | − 3.103 |
| Pd | 0.742 | 0.734 | − 2.010 |
| Au | 0.733 | 0.712 | − 1.957 |
| Ag | 0.647 | 0.499 | − 1.445 |

Dispersion effect alone predicts Volmer–Weber growth (S^d^ < 0) forming isolated particles for all four metals on as-printed GaO_x_.

## ii) Oxygen-vacancy chemisorption effect (ΔW_vac_)

Adding ΔW_vac_ to the dispersion-only spread coefficient


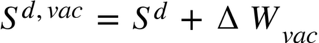


Calculated results are as followed.

| **Metal** | **θ**^[3]^ | **D₀ (eV)**^[4]^ | **ΔW_vac_ (J m⁻²)** | **S^d,vac^** |
| --- | --- | --- | --- | --- |
| Pt | 0.42 | 4.01 | 1.80 | − 1.403 |
| Pd | 0.42 | 3.91 | 1.75 | − 0.26 |
| Au | 0.42 | 2.27 | 1.02 | − 0.937 |
| Ag | 0.42 | 2.25 | 1.01 | − 0.435 |

Adding oxygen-vacancy chemisorption effect still predicts Volmer–Weber growth (S^d,vac^ < 0) forming isolated particles for all four metals on as-printed GaO_x_.

## iii) Alloy-anchoring effect (Δγ_alloy_)

Calculated results are as followed.

| **Alloy** | **ΔH_f_ (kJ mol⁻¹ atoms)** | **Δγ_alloy_ (J m⁻²)** |
| --- | --- | --- |
| Pt-Ga | − 185 | + 2.05 |
| Pd-Ga | − 133 | + 1.47 |
| Au-Ga | − 69.5 | + 0.77 |
| Ag-Ga | − 2.41 | + 0.027 |

The values are DFT calculated value from Materials Project.^[11,12]^ To calculate the Δγ_alloy_ we take μM – μM° from the second point on the Ga-M chemical-potential diagram (Ga + Ga_x_M tie-line) rather than the absolute tangent at μGa = 0. The choice is deliberate and reflects the real interfacial chemistry during sputter deposition.

All four metals display negative formation energies, confirming that their alloying reactions with Ga are exothermic and thermodynamically favorable across the Ga-rich composition range.

Finally, if we add all three effect to estimate the total spreading coefficient (S^total^),


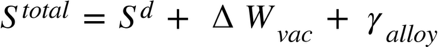


the results are as followed.

| **Metal** | **S^d^** | **ΔW_vac_** | **Δγ_alloy_** | **S^total^** |
| --- | --- | --- | --- | --- |
| Pt | − 3.103 | + 1.80 | + 2.05 | + 0.747 |
| Pd | − 2.010 | + 1.75 | + 1.47 | + 1.21 |
| Au | − 1.957 | + 1.02 | + 0.77 | − 0.167 |
| Ag | − 1.445 | + 1.01 | + 0.027 | − 0.408 |

Adding all three energy terms flips the calculated spreading coefficient S from negative to slightly positive for Pt and Pd, while it remains negative for Au and Ag. This sign change is consistent with our observations that Pt and Pd already form continuous, percolating networks at sub-nanometer thicknesses, whereas Au and Ag do not. The calculation simply demonstrates that oxygen-vacancy chemisorption plus metallic anchoring greatly improves wetting on GaOₓ, especially for Pt and Pd.

Although the calculation well matches with the experimental results in this paper, note that the gap likely could arise due to factors such as non-uniform vacancy and Ga-inclusion distributions, lattice-strain penalties, limited ad-atom mobility, transient local temperature spikes, and other deposition-specific conditions.

2. Supplementary Figures

**Figure S1.** Cross-sectional HAADF-STEM image of the 1 nm 2D Pt film on GaO_x_. The image contrast was adjusted to clearly visualize the atomic lattice fringes of Pt, revealing its crystalline domains and continuous morphology.


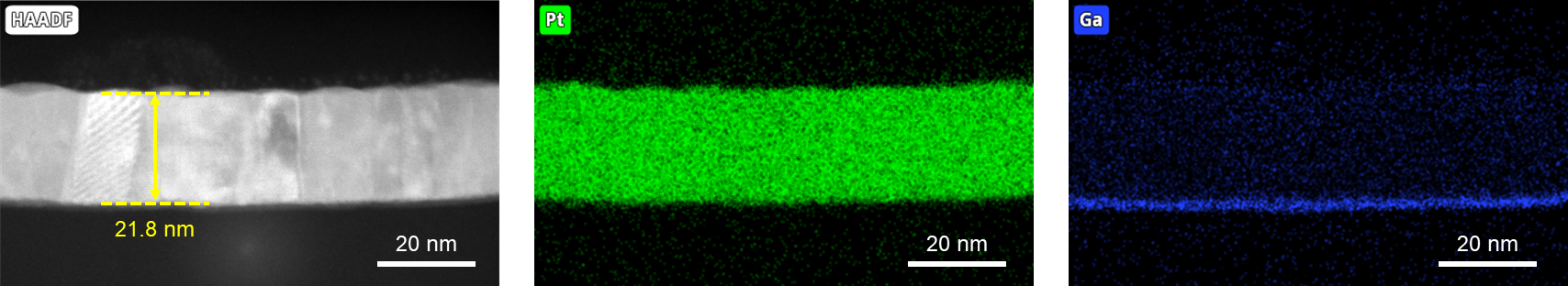


Figure S2. Cross-sectional STEM-HAADF and EDS images of the 20 nm-thick sputtered Pt on GaO_x_. It confirms that its physical thickness matches the value measured by the QCM.


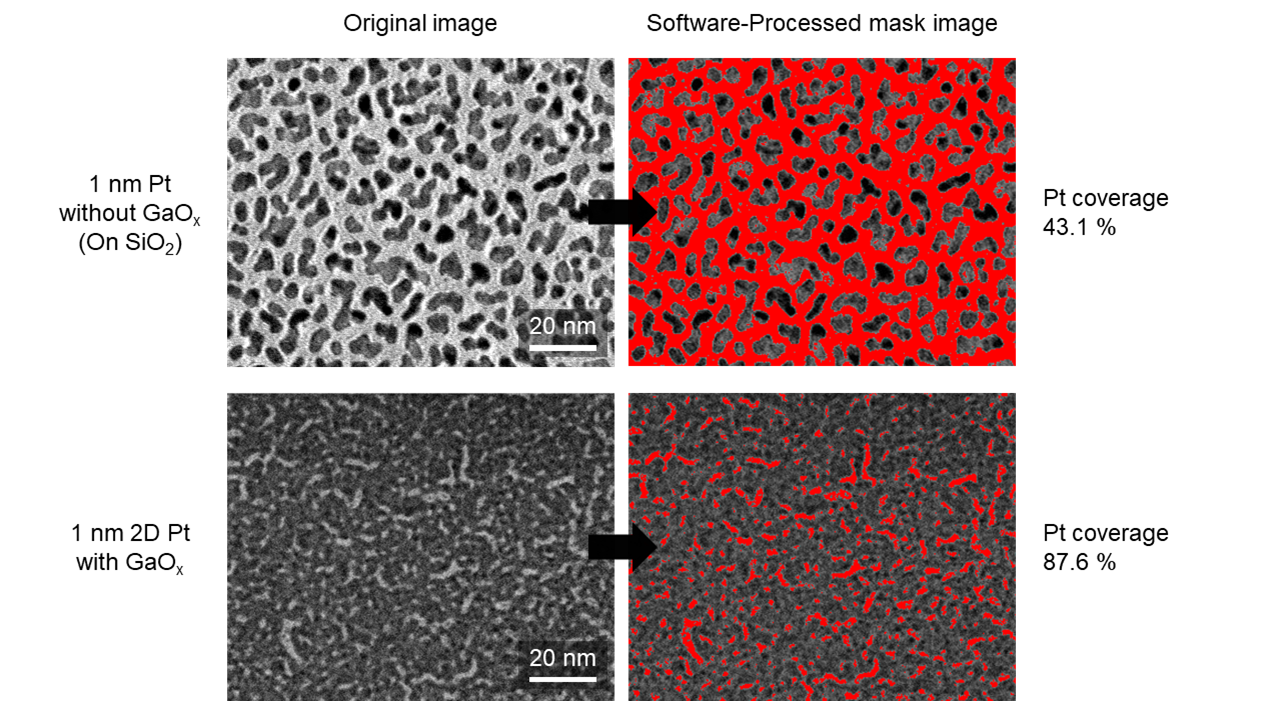


**Figure S3.** Pt surface coverage calculation, with and without GaO_x_ using software-processed mask image. Pt coverage is measured using the software ImageJ by making a mask of empty spaces. The values are averages of measurements taken at multiple spots across multiple samples.


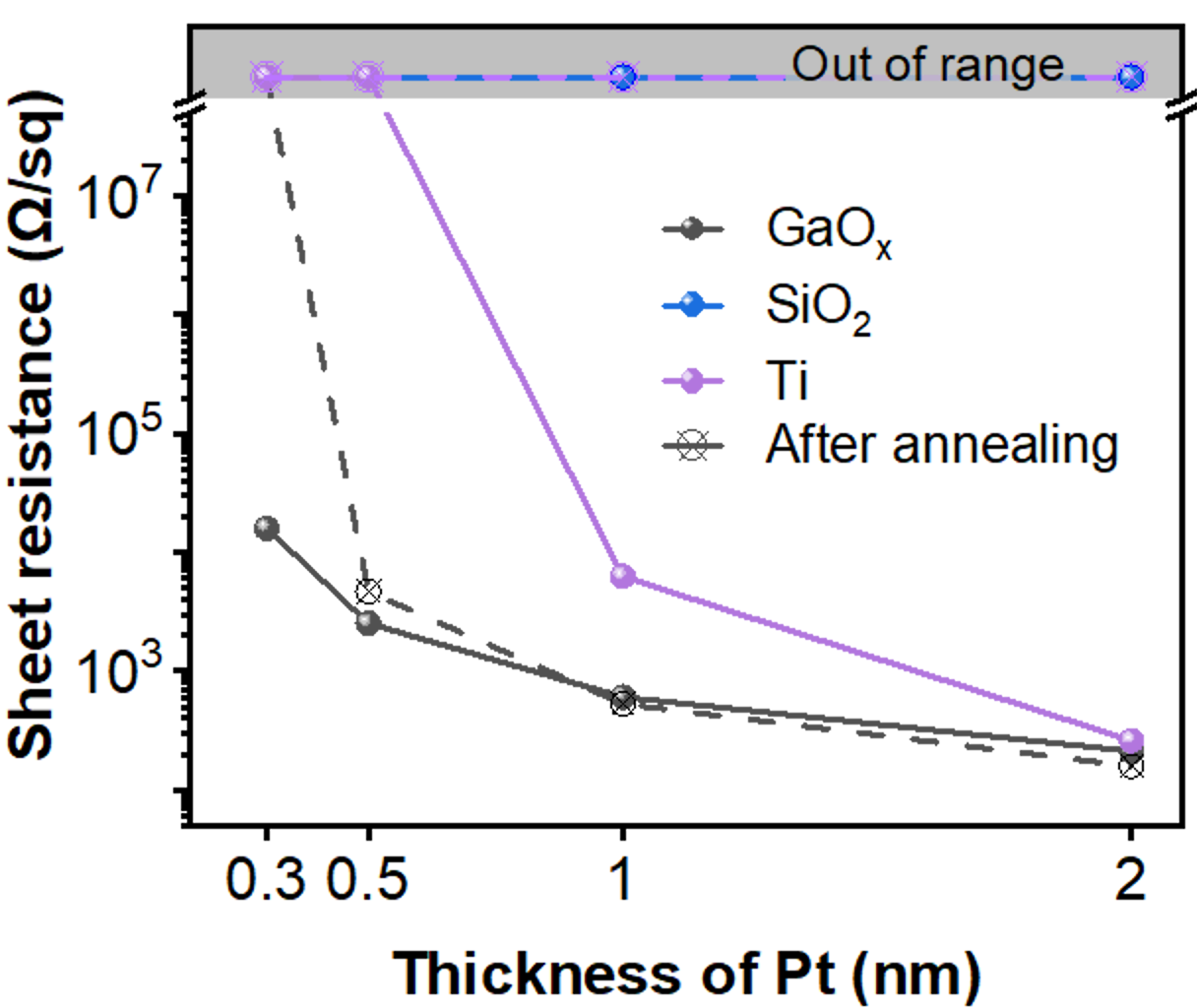


**Figure S4.** Sheet resistance data for Pt film on GaO_x_, SiO_2_, and Ti adhesion layers. Measurements were performed before and after annealing at 600 °C for 1 h. Pt/GaO_x_ shows measurable conductivity at sub-nanometer thickness, while Pt/Ti remains insulating below 2 nm.


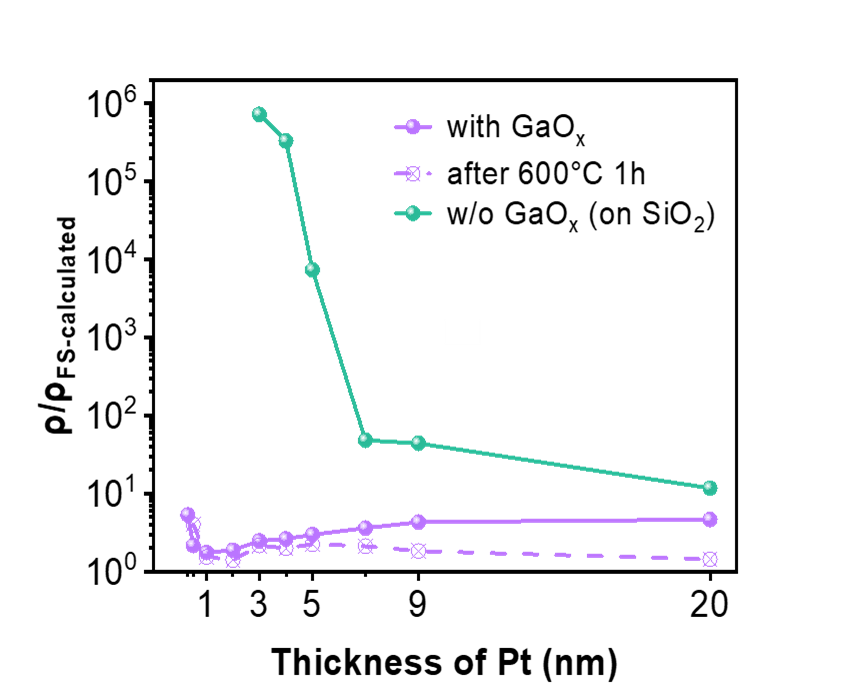


**Figure S5.** Comparison of thickness-dependent resistivity of Pt films with the Fuchs–Sondheimer (FS) model, showing that the observed behavior is governed by conventional size-effect scattering rather than by anomalous transport mechanisms.


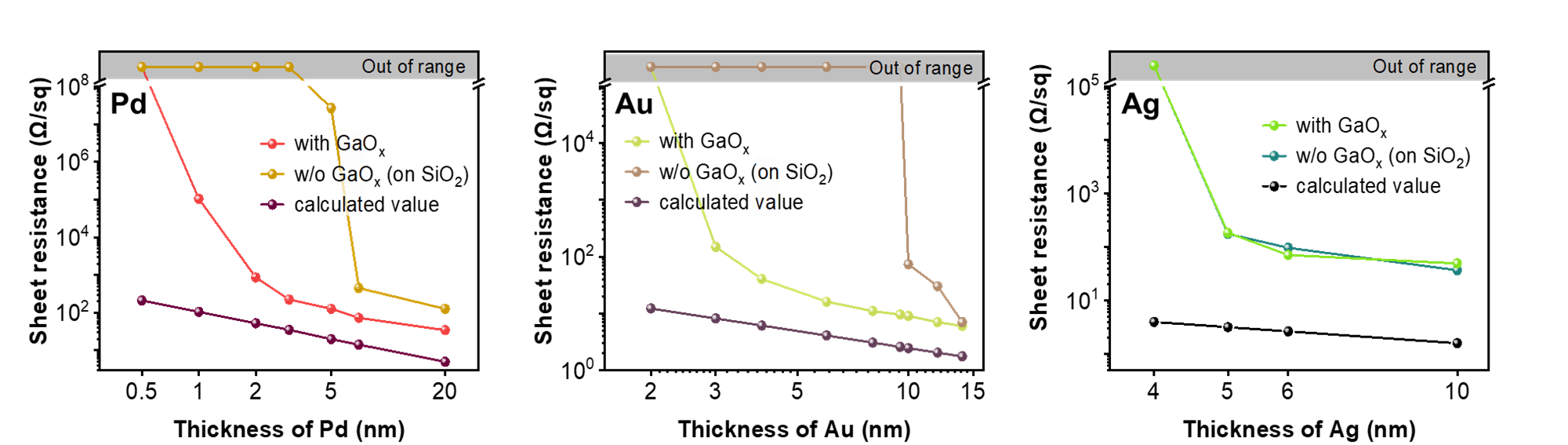


Figure S6. Sheet resistance data for other noble metals (Pd, Au, Ag) deposited on GaO_x_. Percolation threshold (measurable resistance) forms at 1, 3, 5 nm for Pd, Au, and Ag respectively. Calculated value here is the bulk resistivity divided by its corresponding thickness, without considering the quantum confinement effect.

**
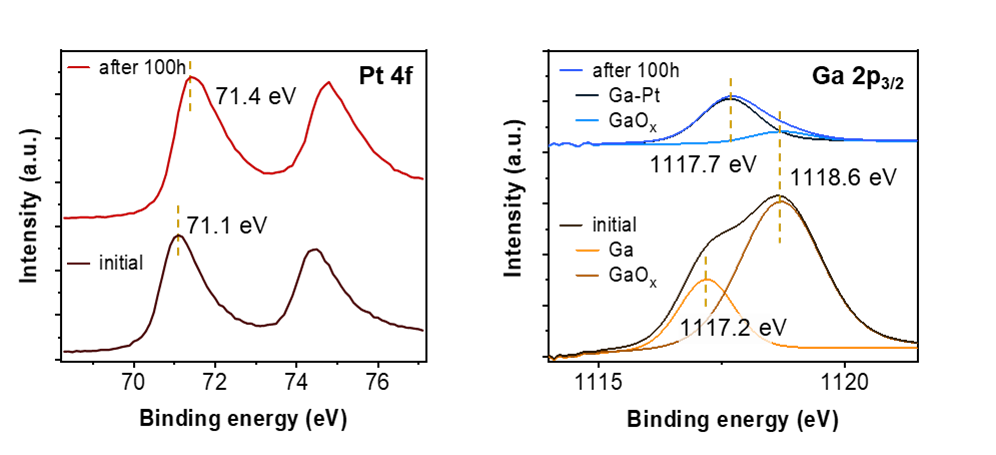
**

**Figure S7.** XPS spectra before and after HER for 100h. The Pt 4f peak reveal a + 0.3 eV shift consistent with Ga-Pt alloy formation. The Ga 2p_3/2_ is deconvoluted into metallic, and oxide components. The oxide peak at 1118.6 eV drastically decreased presenting etching/reduction without noticeable shift. The metallic component peak shifts + 0.5 eV after 100h reaction further confirming a formation of Ga-Pt alloy.


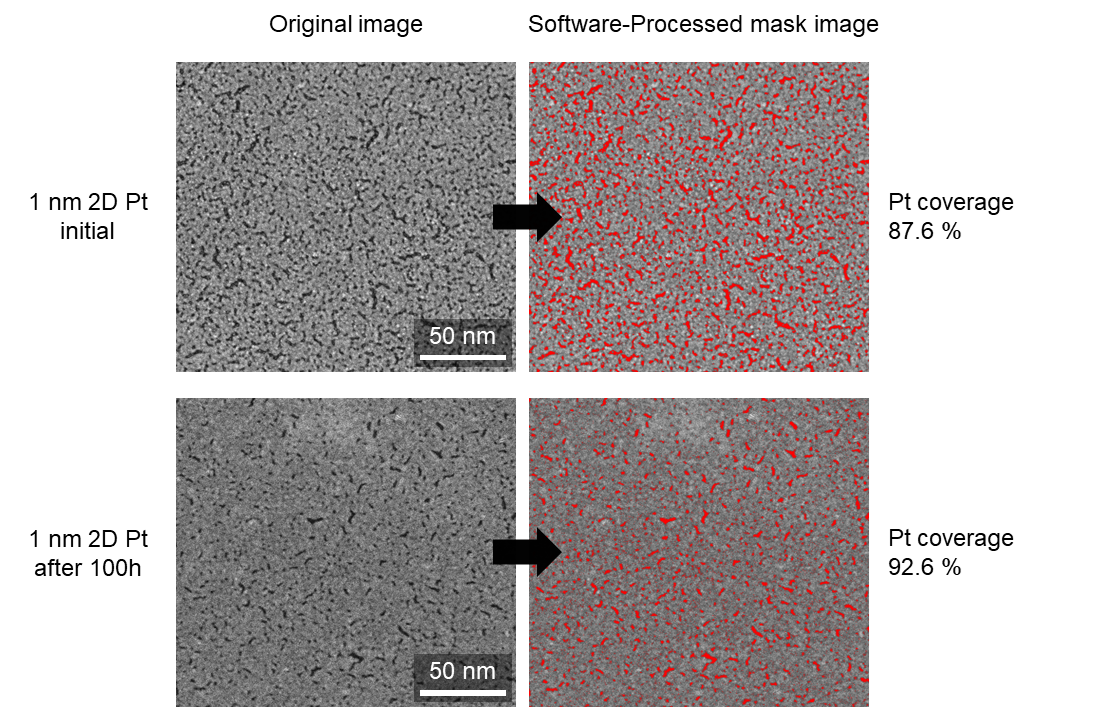


**Figure S8.** Pt surface coverage calculation, before and after 100h HER, using software-processed mask image. Pt coverage is measured using the software ImageJ by making a mask of empty spaces. The values are averages of measurements taken at multiple spots across multiple samples.


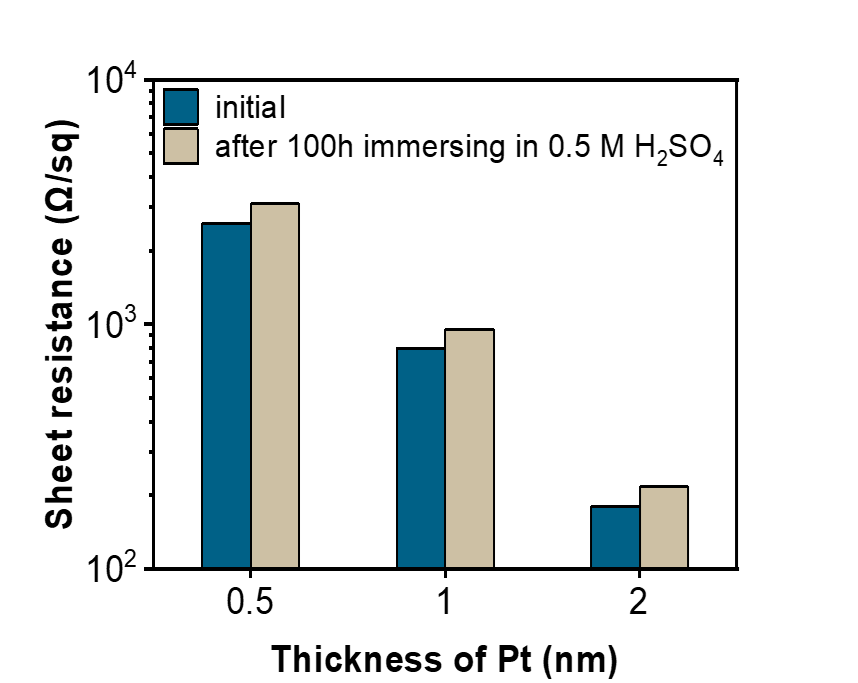


**Figure S9.** Sheet resistance of 2D Pt in 0.5 M H_2_SO_4_ for 100 h under open-circuit condition. Only a slight increase in sheet resistance was observed after immersion, likely caused by minor Pt loss accompanying the dissolution of exposed GaO_x_, and thus the film still exhibits excellent chemical resistance.


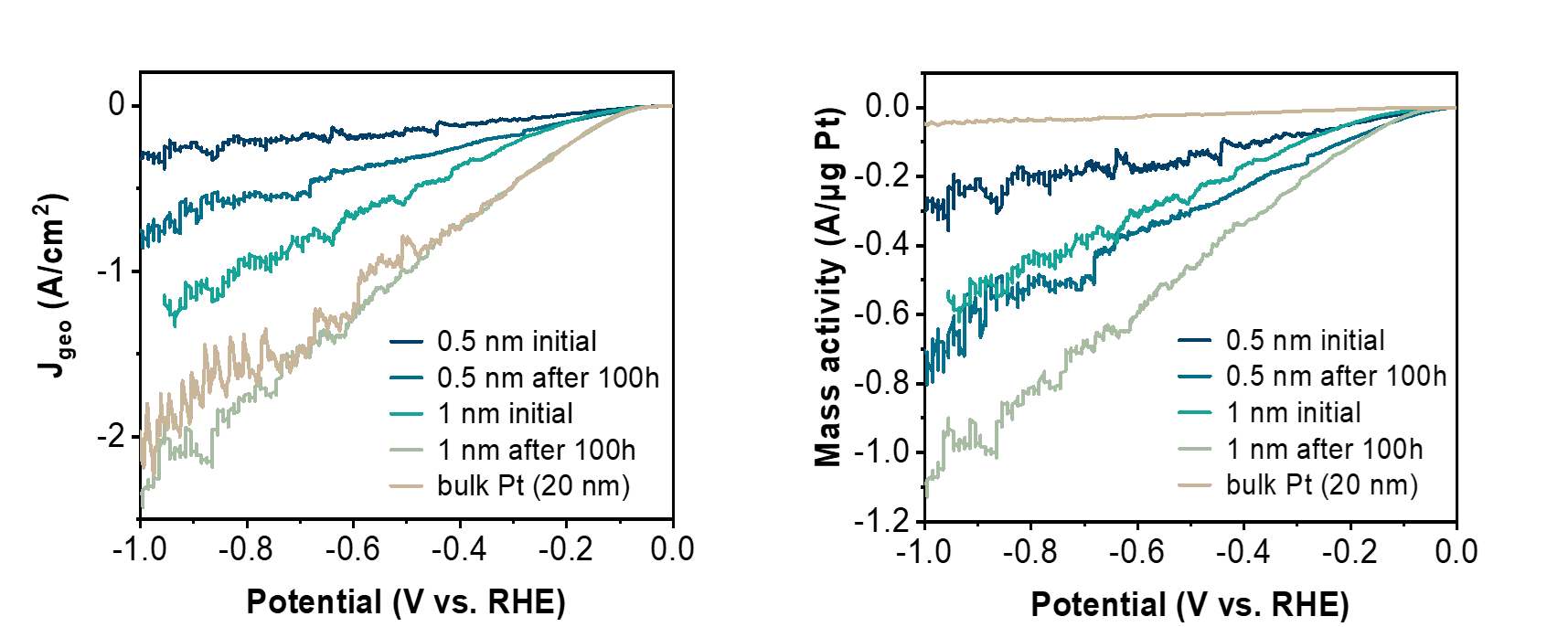


**Figure S10.** Linear sweep voltammograms (LSV) of 2D Pt normalized by (left) geometric area (J_geo_) and (right) Pt mass loading (mass activity, A µg^-1^ Pt).


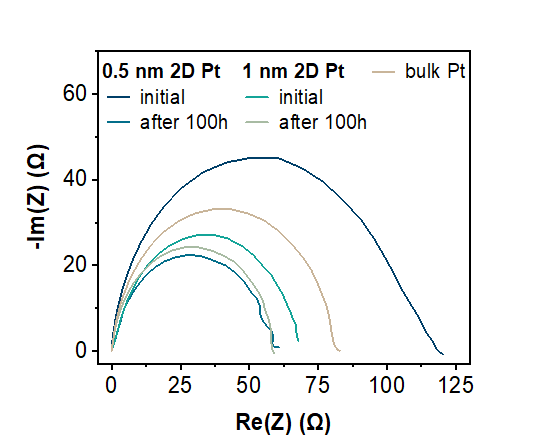


**Figure S11.** Nyquist plot for tested samples. Electrochemical Impedance Spectroscopy (EIS) Nyquist plot is obtained for 0.5 nm 2D Pt, 1 nm 2D Pt, and bulk Pt electrodes (see the legends). It is measured in 0.5 M H_2_SO_4_ at 25 °C. It is obtained at -0.05 V vs RHE with a 10 mV (rms) sinusoidal perturbation. Negligible Warburg-type behavior confirms that mass transport resistance was insignificant under these conditions.


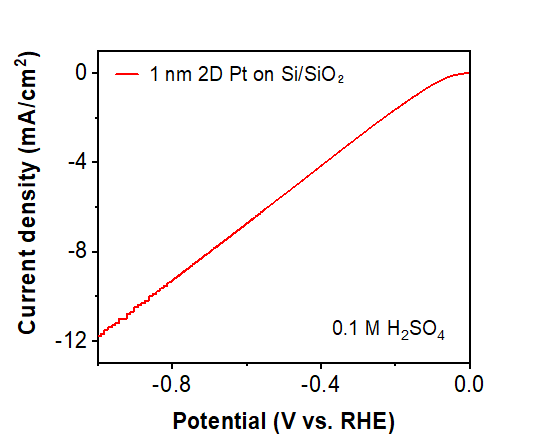


**Figure S12.** LSV of 1 nm 2D Pt on Si/SiO_2_ substate. The Si/SiO_2_ substrate is electrically insulating (350 nm thick SiO_2_), which makes electron transport for the HER occurs exclusively through the 2D Pt layer. Although the high resistance due to the thickness limits the overall reaction, it clearly shows the stable HER.

3. Movies

The supplementary Movie files are provided separately.

Movie S1. Wipe test with 1 nm Pt on sapphire substrate with, and without GaO_x_ adhesion layer.

Movie S2. Free-standing 1 nm 2D Pt with GaO_x_ transferring to TEM grid.

Movie S3. Transparent 2D Pt HER demonstration on ITO substrate.

**4. References**

1. W. D. Kaplan, D. Chatain, P. Wynblatt, and W. C. Carter, “A Review of Wetting versus Adsorption, Complexions, and Related Phenomena: The Rosetta Stone of Wetting,” *Journal of Materials Science* 48, 5681–5717 (2013).

2. M. W. Finnis, “The Theory of Metal-Ceramic Interfaces,” *Journal of Physics: Condensed Matter* 8, 5811 (1996).

3. M. Kong, M. H. Vong, M. Kwak, et al., “Ambient Printing of Native Oxides for Ultrathin Transparent Flexible Circuit Boards,” *Science* 385, 731–737 (2024).

4. J. B. Pedley and E. M. Marshall, “Thermochemical Data for Gaseous Monoxides,” *Journal of Physical and Chemical Reference Data* 12, 967–1031 (1983).

5. L. Z. Mezey and J. Giber, “The Surface Free Energies of Solid Chemical Elements: Calculation from Internal Free Enthalpies of Atomization,” *Japanese Journal of Applied Physics* 21, 1569 (1982).

6. W. Jung, M. H. Vong, K. Kwon, et al., “Giant Decrease in Interfacial Energy of Liquid Metals by Native Oxides,” *Advanced Materials* 36, 2406783 (2024).

7. T. Jiang, H. Wang, H. Zhu, et al., “Single-Crystalline β-Ga2O3 Homoepitaxy on a Near van der Waals Surface of (100) Substrate,” *Advanced Science* 12, 2417436 (2025).

8. S. R. Narayan, J. M. Day, H. L. Thinakaran, et al., “Comparative Study of Surface Energies of Native Oxides of Si(100) and Si(111) via Three Liquid Contact Angle Analysis,” *MRS Advances* 3, 3379–3390 (2018).

9. C. V. Thompson, “Solid-State Dewetting of Thin Films,” *Annual Review of Materials Research* 42, 399–434 (2012).

10. S. Blackwell, R. Smith, S. D. Kenny, and J. M. Walls, “Modeling Evaporation, Ion-Beam Assist, and Magnetron Sputtering of Thin Metal Films over Realistic Time Scales,” *Physical Review B* 86, 035416 (2012).

11. S. P. Ong, L. Wang, B. Kang, and G. Ceder, “Li-Fe-P-O2 Phase Diagram from First Principles Calculations,” *Chemistry of Materials* 20, 1798–1807 (2008).

12. A. Jain, et al., “Commentary: The Materials Project: A Materials Genome Approach to Accelerating Materials Innovation,” *APL Materials* 1, 011002 (2013).
